# Risk factors of interventional radiology/surgery for colonic diverticular bleeding

**DOI:** 10.1002/jgh3.12499

**Published:** 2021-01-27

**Authors:** Yoshinori Sato, Hiroshi Yasuda, Yusuke Nakamoto, Hirofumi Kiyokawa, Masaki Yamashita, Yasumasa Matsuo, Tadateru Maehata, Hiroyuki Yamamoto, Hidefumi Mimura, Fumio Itoh

**Affiliations:** ^1^ Division of Gastroenterology and Hepatology, Department of Internal Medicine St. Marianna University School of Medicine Kawasaki Japan; ^2^ Department of Radiology St. Marianna University School of Medicine Kawasaki Japan

**Keywords:** colonic diverticular bleeding, interventional radiology, surgery

## Abstract

**Background and Aim:**

Colonic diverticular bleeding (CDB) stops spontaneously, but sometimes, excessive bleeding does not allow hemostasis and requires interventional radiology (IR)/surgery. We examined risk factors in patients who required IR/surgery for CDB and late recurrent bleeding rate after IR/surgery.

**Methods:**

This retrospective case–control study was conducted at a tertiary center. We included 608 patients who required hospitalization for CDB. Patients were investigated for risk factors using logistic regression analysis. We also investigated early and late recurrent bleeding rates following IR/surgery.

**Results:**

In 261 patients (42.9%), the bleeding source was identified, and endoscopic hemostasis was performed; 23 (3.8%) required IR/surgery. In multivariate analysis, shock state with a blood pressure of ≤90 mmHg (*P* < 0.001; odds ratio [OR], 20.1; 95% confidence interval [CI], 5.08–79.5), positive extravasation on contrast‐enhanced computed tomography (*P* < 0.001; OR 9.5, 95% CI 2.85–31.4), two or more early recurrent bleeding episodes (*P* = 0.002; OR 7.4, 95% CI 2.14–25.4), and right colon as the source of bleeding (*P* = 0.023; OR 4.1, 95% CI 1.25–14.0) were independent risk factors requiring IR/surgery. Early recurrent bleeding was observed in 0% and 28.0% patients (*P* < 0.001) in the IR/surgery and no IR/surgery groups, respectively, whereas late recurrent bleeding rate was observed in 43.4% and 30.7% patients (*P* = 0.203) in the IR/surgery and no IR/surgery groups, respectively. Four patients who required surgery experienced late recurrent bleeding at a site different from the initial CDB.

**Conclusions:**

Although IR/surgery is an effective hemostatic treatment wherein endoscopic treatment is unsuccessful, late recurrent bleeding cannot be prevented.

## Graphical abstract

23 (3.8%) colonic diverticular patietns required interventional radiology/surgery. We examined risk factors of interventional radiology/surgery of colonic diverticular bleeding. Although interventional radiology/surgery is an effective treatment, late recurrent bleeding cannot be prevented.

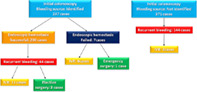

## Introduction

Colonic diverticular bleeding (CDB) is the most common cause of acute lower gastrointestinal bleeding (LGIB).^1^, ^2^ LGIB can be serious and potentially lead to death.^3^, ^4^, ^5^ In 70–90% of patients, CDB stops bleeding spontaneously;^6^, ^7^, ^8^ however, some patients develop active bleeding that requires hemostasis. Colonoscopy is able to achieve diagnosis and treatment; therefore, it plays an important role in LGIB. In case of CDB, endoscopic hemostasis is recommended because the adverse events are limited for colonoscopy.^9^, ^10^, ^11^ The clip method was the mainstream endoscopic hemostasis,^12^ but in recent years, the use of ligation such as endoscopic band ligation (EBL)^13^ and detachable snare method^14^ have been reported. However, despite the progress in endoscopic hemostasis methods, there are cases wherein endoscopic hemostasis is difficult, and interventional radiology (IR) and surgery are required.^9^, ^11^, ^15^, ^16^, ^17^, ^18^ According to the Japanese Guidelines for CBD and colonic diverticulitis, IR or surgery is recommended when endoscopic hemostasis is unsuccessful, and bleeding continues.^19^ Ishii et al. reported that, because the rate of success for endoscopic hemostasis in the ascending colon is low, the required rate of IR is high.^15^ Wong et al. also reported that surgery for CDB was required more often for the right colon than for the left colon.^18^ However, limited reports have examined the risk factors for IR/surgery in CDB cases.^15^, ^18^ Thus, in the present study, we aimed to examine the risk factors for IR/surgery in CDB cases that required IR/surgery.

## Methods

### *Study design*

This was a retrospective case–control study conducted at a tertiary center in accordance with the Declaration of Helsinki. The local ethics committee of our hospital approved the study protocol. All authors had access to study data and approved the final draft of the manuscript.

### *Patients and data collection*

We included patients aged ≥18 years who required hospitalization for CDB between January 2004 and May 2019. Diagnostic criteria for CDB were as follows: (i) active bleeding, nonbleeding visible vessel, or adherent clot from specific diverticulum that was confirmed on colonoscopy,^20^, ^21^ and (ii) although the source of bleeding was not identified using colonoscopy, there is no lesion that could cause bloody stool in sites other than the diverticulum. In addition, computed tomography (CT) and esophagogastroduodenoscopy excluded upper gastrointestinal and small intestinal bleeding.

We investigated gender, average age, comorbidities, and medications of the hospitalized cases. We also examined the vital signs, positive rate of extravasation on contrast‐enhanced CT, rate of identification for the source of bleeding, rate of endoscopic hemostasis, transfusion volume, and rates of early and late recurrent bleeding. Patient data were retrospectively collected using the endoscopy database NEXUS (FUJIFILM Holdings Co., Tokyo, Japan) and electronic medical records. Data on late recurrent bleeding were obtained through phone calls. Shock was defined as a blood pressure of ≤90 mmHg caused by bleeding. Early recurrent bleeding was defined as fresh bloody stool that occurred within 30 days of hospitalization, dark red stool that required another emergency colonoscopy, or a decrease in hemoglobin to ≤2 g/dL in blood test. Late recurrent bleeding was defined as hospitalization being required for CDB ≥31 days after the initial admission. Patients on hemodialysis or peritoneal dialysis or those with a glomerular filtration rate of <60 mL/min were considered to have chronic kidney disease.

### *Response to* *CDB* *upon admission*

Upon admission, contrast‐enhanced CT was performed for all patients as long as there was no history of kidney failure or allergy to the contrast agent. Colonoscopy was also performed for all patients during hospitalization. Bowel irrigation solution containing polyethylene‐glycol (PEG) was ingested before colonoscopy was performed. However, if contrast‐enhanced CT confirmed positive extravasation, and persistent bleeding was suspected, emergency colonoscopy was performed without PEG based on the judgment of the attending physician.

PCF‐Q260AZI or PCF‐290AZI (Olympus Co., LTD, Tokyo, Japan) was used for colonoscopy. According to Jensen et al.,^20^, ^21^ we performed endoscopic hemostasis when it was determined to be stigmata of a recent hemorrhage. For endoscopic hemostasis, the clip method or EBL method was performed based on the judgment of the endoscopists. HX‐610‐135 or HX‐610‐135S (Olympus Co., LTD, Tokyo, Japan) was used for the clip method. If endoscopic hemostasis was difficult to perform due to massive bleeding or repeated recurrent bleeding, IR or surgery was performed based on the judgment of the attending physician.

### *Outcomes*

The primary outcome was the rate of IR/surgery and risk factors. The secondary outcome was early and late recurrent bleeding after IR/surgery.

### *Statistical analysis*

Results are presented as median and average for continuous variables. *χ* ^2^‐test and Fisher's exact test were used to compare categorical data, and Student's *t*‐test and Mann–Whitney's *U* test were used to compare continuous data. Logistic regression analysis was performed to the calculate odds ratio (OR) of risk factors for IR and surgery for CDB. Multivariate analysis was performed for risk factors that showed statistical significance in univariate analysis. Comparison of the recurrence‐free survival period was performed using a log‐rank test with the Kaplan–Meier method. A *P*‐value of <0.05 was considered to indicate statistical significance. Statistical analyses were performed using SPSS (version 22.0; IBM Corp., Armonk, NY, USA).

## Results

### *Patient characteristics*

The clinical characteristics of patients are summarized in Table 1. During the study period, 608 subjects (male: 415, female: 193, average age: 72.4 ± 13.0 years) were hospitalized for CDB. The most common underlying condition was hypertension in 384 patients (63.1%). A total of 248 patients (40.8%) were administrated antithrombotic agents. The source of bleeding was identified for 261 patients (42.9%). For endoscopic hemostasis, the clip method was used for 36.2%. For 19 patients, endoscopic hemostasis failed, and IR was required (3.1%), and 4 patients required surgery (0.7%). At the time of admission, 92 patients (15.1%) were in a state of shock, and 149 patients (24.5%) were positive for extravasation on contrast‐enhanced CT. The average transfusion volume was 1.4 ± 2.9 units.

**Table 1:** Clinical characteristics of patients

Characteristics		*n* = 608
Gender	Male	415 (68.2%)
	Female	193 (31.8%)
Age	Average ± SD	72.4 ± 13.0
Comorbidities	Hypertension	384 (63.1%)
	Diabetes mellitus	102 (16.7%)
	Chronic kidney disease	232 (38.1%)
	Cardiovascular disease	181 (30.0%)
	*Cerebrovascular disease*	72 (11.8%)
	Respiratory disease	21 (3.5%)
	Liver cirrhosis	6 (1.9%)
	Malignant disease	47 (7.7%)
Medication	Antithrombotic agents	248 (40.8%)
	Aspirin	162 (26.6%)
	Thienopyridine	51 (8.4%)
	Warfarin	51 (8.4%)
	DOACs	30 (4.9%)
	DAPT	35 (5.8%)
Bleeding source	NSAIDs Detection rate Right colon Left colon	196 (32.2%) 261 (42.9%) 177 (29.1%) 84 (13.8%)
Hemostasis method	Hemoclip Band ligation Interventional radiology Surgery	220 (36.2%) 18 (3.0%) 19 (3.1%) 4 (0.7%)
Shock	BP < 90 mmHg	92 (15.1%)
Computed tomography extravasation	Positive	149 (24.5%)
Blood transfusion	Average units (± SD)	1.4 ± 2.9

BP, blood pressure; DAPT, dual antiplatelet therapy; DOACs, direct oral anticoagulants; NSAIDs, non‐steroidal anti‐inflammatory drugs.

**Figure 1:**
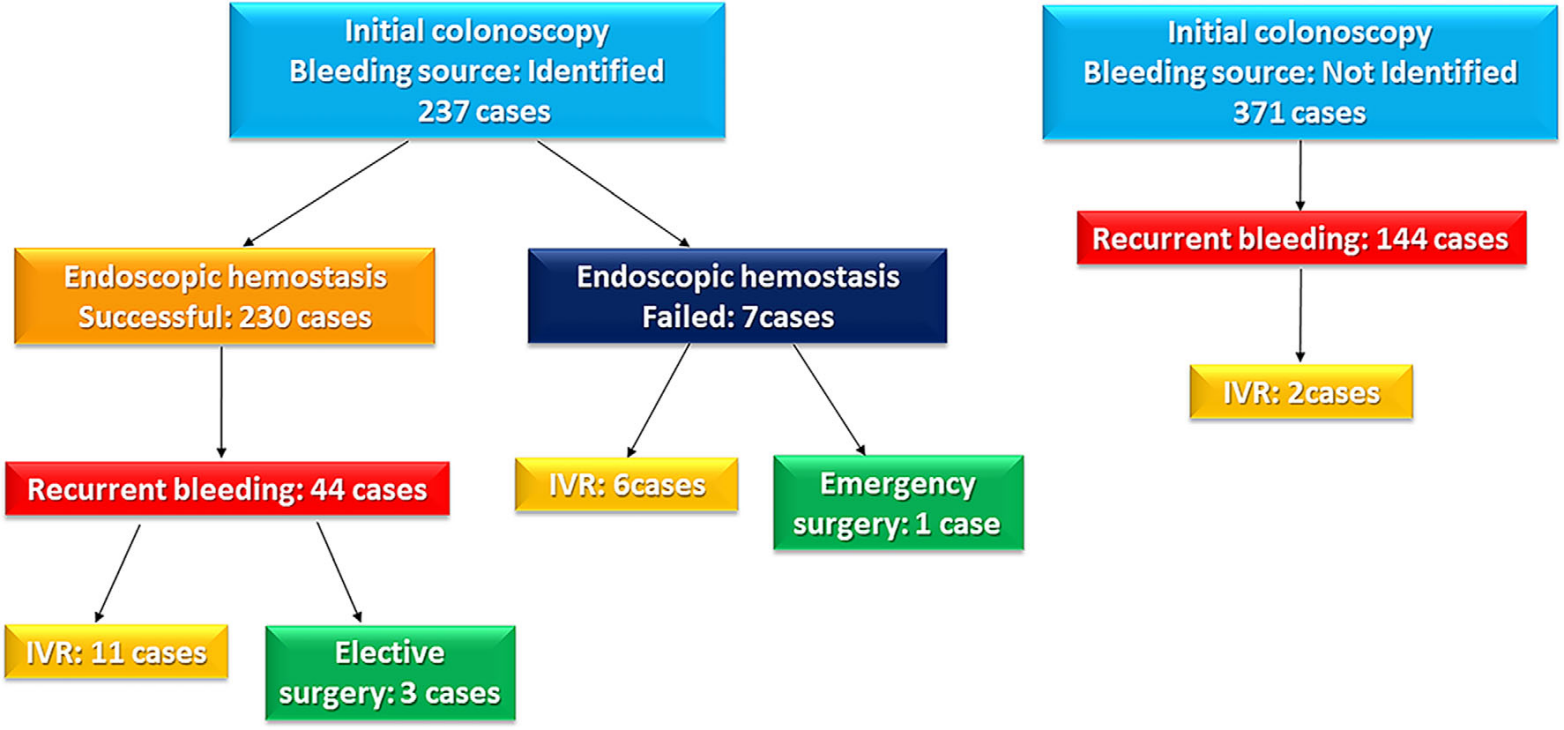
Study flow diagram of patients who underwent interventional radiology and surgery for colonic diverticular bleeding.

Figure 1 shows the timing of IR/surgery for patients who required them. Colonoscopy was performed for all patients after admission, and initial colonoscopy identified the source of bleeding in 237 patients. Among these, emergency IR was performed for six patients, and emergency surgery was performed for one patient because initial endoscopic hemostasis failed with persistent bleeding. Endoscopic hemostasis was temporarily successful for 230 patients; however, IR was performed for 11 patients, and elective surgeries were performed for three patients because of repeated recurrent bleeding. For 371 patients, the source of bleeding could not be identified on initial colonoscopy; of these, 144 patients experienced recurrent bleeding. Of these, two patients required IR because of repeated recurrent bleeding.

### *Primary outcome*

In patients who required IR/surgery, the risk factors were analyzed and are given in Table 2. There were 23 patients (3.8%) who required IR/surgery. Univariate analysis showed that the use of antithrombotic drugs (*P* = 0.045), right colon as the bleeding source (*P* < 0.001), two or more early recurrent bleeding episodes (*P* < 0.001), state of shock with a blood pressure of ≤90 mmHg at the time of admission (*P* < 0.001), positive extravasation on contrast‐enhanced CT (*P* < 0.001), and four units or more for transfusion (*P* < 0.001) showed statistically significant differences. In multivariate analysis, the state of shock (*P* < 0.001; OR of 20.09, 95% confidence interval [CI] of 5.08–79.46), positive extravasation on contrast‐enhanced CT (*P* < 0.001; OR 9.47, 95% CI 2.85–31.44), two or more episodes of early recurrent bleeding (*P* = 0.002; OR 7.38, 95% CI 2.14–25.44), and right colon as the source of bleeding (*P* = 0.023; OR 4.11, 95% CI 1.21–13.97) were independent risk factors for IR/surgical treatment.

**Table 2:** Independent risk factors that required interventional radiology/surgery using logistic regression analysis

	Univariate analysis			Multivariate analysis	
Variables	IR/surgery (*n* = 23)	Without IR/surgery (*n* = 585)	*P*‐value	OR (95% CI)	*P*‐value
Age ≥70	17 (73.9%)	375 (64.1%)	0.383		
Gender					
Male	20 (87.0%)	395 (67.5%)	0.065		
Female	3 (13.0%)	190 (32.5%)			
Comorbidities					
Hypertension	10 (43.4%)	365 (62.3%)	0.080		
Diabetes mellitus	6 (26.1%)	96 (16.4%)	0.251		
Chronic kidney disease	8 (34.8%)	224 (38.3%)	0.829		
Cardiovascular disease	10 (43.5%)	171 (29.2%)	0.164		
Cerebrovascular disease	14 (17.4%)	68 (11.6%)	0.337		
Malignant disease	1 (4.3%)	46 (7.8%)	0.715		
Medication					
Antithrombotic agents	14 (60.8%)	234 (40.0%)	0.045	2.538 (0.804–8.017)	0.112
Aspirin	9 (39.1%)	155 (26.4%)	0.889		
Thienopyridine	3 (13.0%)	48 (8.2%)	0.430		
Warfarin	3 (13.0%)	48 (8.2%)	0.430		
DOACs	3 (13.0%)	37 (6.3%)	0.187		
DAPT	3 (13.0%)	32 (5.4%)	0.140		
NSAIDs	10 (43.5%)	186 (31.7%)	0.258		
Bleeding source					
Right colon	18 (78.2%)	159 (27.1%)	<0.001	4.119 (1.215–13.97)	0.023
Left colon	5 (21.7%)	79 (13.5%)			
Recurrent bleeding >2 times	11 (47.8%)	69 (11.8%)	<0.001	7.384 (2.143–25.44)	0.002
Shock BP <90 mmHg	16 (69.6%)	76 (13.0%)	<0.001	20.09 (5.080–79.46)	<0.001
Positive CT extravasation	17 (73.9%)	132 (22.6%)	<0.001	9.467 (2.851–31.44)	<0.001
Blood transfusion >4 units	14 (60.9%)	93 (15.9%)	<0.001	1.809 (0.534–6.133)	0.341

BP, blood pressure; CI, confidence interval; DAPT, dual antiplatelet therapy; DOACs, direct oral anticoagulants; IR, interventional radiology; NSAIDs, non‐steroidal anti‐inflammatory drugs; OR, odds ratio.

### *Secondary outcome*

The results of early and late recurrent bleeding rates following IR/surgery are shown in Table 3. Early recurrent bleeding rate was 0 for the group that underwent IR/surgery, whereas it was 28.0% for the group that did not undergo IR/surgery; it was significantly lower for the group that underwent IR/surgery (*P* < 0.001). Late recurrent bleeding rate was 43.4% and 30.7% for the groups that underwent and did not undergo IR/surgery, respectively; there was no statistically significant difference between these groups (*P* = 0.203). The median time to late bleeding was 8 and 12 months for the groups that underwent and did not undergo IR/surgery, respectively (*P* = 0.387). The median follow‐up period was 40 and 33 months for the groups that underwent and did not undergo IR/surgery, respectively (*P* = 0.412); no statistically significant difference was observed.

**Table 3:** Early recurrent bleeding and late recurrent bleeding rate

	IR/surgery (*n* = 23)	Without IR/surgery (*n* = 585)	*P‐*value
Early recurrent bleeding rate	0 (%)	164 (28.0%)	<0.001
Period until recurrent bleeding (median, range: days)	—	2 (0–23)	—
Recurrent bleeding times (median, range)	—	1 (1–6)	—
Late recurrent bleeding rate	10 (43.4%)	181 (30.7%)	0.203
Period until recurrent bleeding (median, range: months)	8 (2–144)	12 (1–146)	0.387
Recurrent bleeding times (median, range)	2 (1–4)	1 (1–10)	0.837
Follow‐up period (median, range: months)	40 (7–144)	33 (1–207)	0.412

IR, interventional radiology.

**Figure 2:**
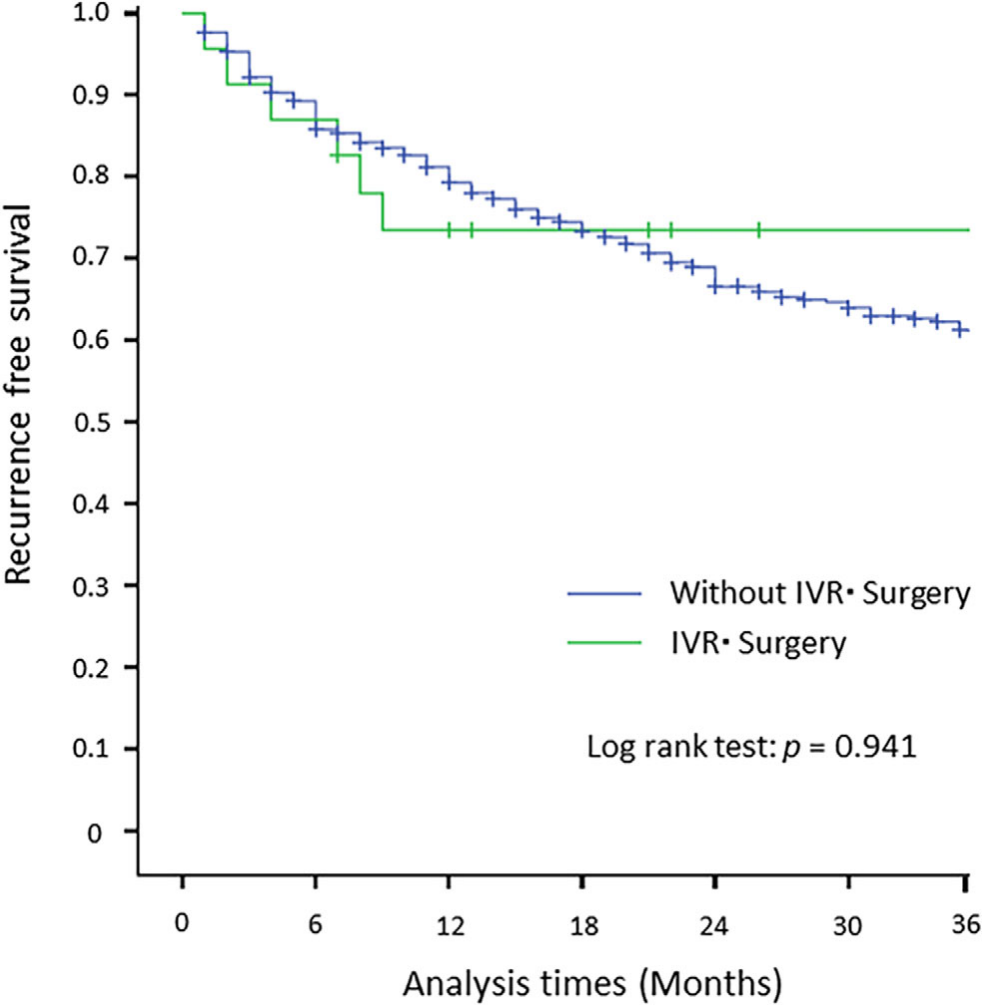
Recurrence‐free survival period of patients who needed and did not need interventional radiology/surgery.

The recurrence‐free survival rate based on the Kaplan–Meier method is shown in Figure 2. During the 3 years of follow‐up survey, recurrence‐free survival rates at 1 and 3 years were 73.9% and 69.6% for the IR/surgery group and 84.3% and 73.7% for the group without IR/surgery, respectively, not showing statistically significant difference (log‐rank test: *P* = 0.941).

The details of IR/surgery cases are shown in Table 4. The success rate of IR was 18 of 19 cases (94.4%), wherein superselective embolization was not possible for one patient, and only angiography was performed. Postembolization complications were not observed in any patient. Following embolization, late recurrent bleeding was confirmed in six patients (31.6%).

**Table 4:** Details of interventional radiology/surgery cases

Gender	Age	Location	IR/surgery	Endoscopic hemostasis method	Initial endoscopic hemostasis	Complication	Duration until late recurrent bleeding (months)
M	75	A	IR: NBCA	Clip	Success	None	139
M	75	C	IR: Coil	Clip	Fail	None	144
M	44	A	IR: Coil	Clip	Success	None	4
M	82	A	IR: Coil	Clip	Fail	None	2
M	68	T	IR: Coil	Clip	Success	None	7
M	70	S	IR: Only angiography	Clip	Success	None	8
M	81	A	IR: Coil	Clip	Success	None	—
M	76	A	IR: Coil	Clip	Success	None	—
M	80	A	IR: Coil	Clip	Success	None	—
M	52	A	IR: Coil	Clip	Success	None	—
M	71	A	IR: Coil	Clip	Success	None	—
M	78	A	IR: Coil	Clip	Fail	None	—
M	86	A	IR: Coil	EBL	Success	None	—
M	79	A	IR: Coil	EBL	Success	None	—
M	68	C	IR: Coil	Clip	Fail	None	—
M	83	C	IR: Coil	Clip	Fail	None	—
M	80	A	IR: Coil	Clip	Fail	None	—
F	86	A	IR: NBCA/Coil	Clip	Fail	None	—
F	90	D	IR: Coil	Clip	Fail	None	—
M	63	T	Transverse colectomy	Clip	Success	None	2
M	77	S	Simple reefing	Clip	Fail	None	8
F	66	T	Transverse colectomy	Clip	Success	None	39
M	76	T	Transverse colectomy	Clip	Success	None	48

A, ascending colon; C, cecum; D, descending colon; EBL, endoscopic band ligation; F, female; IR, interventional radiology; M, male; NBCA, *N*‐butyl‐2‐cyanoacrylate; S, sigmoid colon; T, transverse colon.

There was no death and complications following emergency or elective surgeries. In the four cases of surgery, late recurrent bleeding occurred in all cases and required hospitalization. In all cases, the source of bleeding of late recurrent bleeding was different from that of the initial bleeding site.

## Discussion

Endoscopic hemostasis for CDB is an effective method for primary hemostasis with a success rate of 88–100%.^12^, ^14^ However, endoscopic hemostasis is difficult for certain cases, and IR or surgery is required.^9^, ^11^, ^15^, ^16^, ^17^, ^18^ Japanese guidelines for CDB recommend that IR be performed for patients with CDB, wherein endoscopic hemostasis is difficult to perform because of massive or persistent bleeding or wherein recurrent bleeding occurs despite endoscopic hemostasis.^19^ In the present study, IR/surgery was required for 23 patients (3.8%) with CDB. Independent risk factors for IR/surgery were shock with a blood pressure of ≤90 mmHg, positive extravasation on contrast‐enhanced CT, two or more episodes of recurrent bleeding, and the right colon as the source of bleeding. In particular, the state of shock was the risk factor with the highest OR. Hemorrhagic shock is a form of hypovolemic shock that is life‐threatening because the loss of blood is ≥30%.^22^ Extravasation is a contrast‐enhanced CT finding observed when the bleeding is ≥0.3 mL/min. ^23^ Both are findings that indicate active bleeding for CDB. According to Doi et al. who studied 142 patients with CDB, in cases of both shock and positive CT extravasation, two of five patients developed severe bleeding who required hemostasis with IR.^24^ In a report by Nakatsu et al. who studied 346 patients with CDB, the rate of bleeding source identification in emergency colonoscopy was 68% for positive extravasation and 20% for negative extravasation, and it was significantly higher for those with positive extravasation.^25^ Shock and positive extravasation strongly indicate massive or active bleeding, and rapid hemostatic treatment is necessary while maintaining the vital signs in an emergency situation. As a method of hemostasis, endoscopic hemostasis is recommended first because the incidence of adverse events from emergency colonoscopy performed on LGIB cases is 0.3–1.3% for >2400 cases, which is safer than IR and surgery.^9^, ^11^ However, when there is persistent massive bleeding, it is difficult to secure the field of view using colonoscopy even after PEG administration. In particular, in cases of CDB, bleeding from the right colon tends to be more severe than from the left colon; furthermore, if endoscopic hemostasis is successful, subsequent recurrent bleeding tends to occur.^15^, ^17^, ^18^, ^26^ Therefore, when bleeding occurs from the right colon, the likelihood of problems with hemostasis along with colonoscopy is significantly higher than the bleeding from the left colon, thus having a high likelihood for IR or surgery.^15^, ^17^, ^18^, ^26^ These reports on factors that make endoscopic hemostasis difficult support the results of the present study. Furthermore, from the present study, we found that, when there are two or more episodes, early recurrent bleeding also becomes an independent risk factor for IR/surgery. The median number of early recurrent bleeding episodes in the present study was one. However, there was a patient with six episodes of early recurrent bleeding. In a previous report, although temporary endoscopic hemostasis could be achieved, the rate of subsequent early recurrent bleeding was high (0–50%).^27^ On occurrence of multiple recurrent bleeding, it is important to consider alternative hemostasis methods, such as IR and surgery.

Recently, the success rate of embolization is reported to be ≥70–90%,^11^, ^28^ and the incidence of intestinal ischemia as a complication is 0–10%.^29^, ^30^ On the other hand, surgery is also an effective hemostasis method if the source of bleeding can be identified. However, there is a report stating that the rate of deaths due to emergency colectomy for CDB is 20%, which is high.^31^ In the present study, the success rate of IR was 18 of 19 cases (94.4%). Excluding one case, wherein the insertion of catheter was difficult due to vessel tortuosity, superselective embolization was possible for all cases. In all the present IR cases, colonoscopy was performed beforehand. The source of bleeding was endoscopically identified for all cases, wherein endoscopic hemostasis with clips was performed in 12 cases, and marking clips were placed in 7 of these. During angiography, clips placed at the site of bleeding worked as markers; thus, superselective catheter insertion at the bleeding site became possible. The existence of these clips might have been why the success rate of embolization was high. The fact that there was no adverse event such as intestinal ischemia following IR might also have been why superselective embolization was possible. Moreover, in the present study, there was only one case where endoscopic hemostasis failed and emergency surgery was performed. This case did not allow the use of a contrast agent due to chronic kidney dysfunction, which made IR impossible. Therefore, after identifying the source of bleeding using colonoscopy, emergency surgery was performed to surgically suture the causal diverticulum.

After IR/surgery, there was no case with early recurrent bleeding, which was significantly lower than cases without IR/surgery. As such, IR/surgery is an effective hemostatic treatment for severe CDB cases. However, there was no statistically significant difference in late recurrent bleeding rate among the present cases with or without IR/surgery. Specifically, in the four cases with surgery, the source of bleeding was identified preoperatively, the bleeding site was resected in three cases, and a causal diverticulum was sutured in one. However, late recurrent bleeding occurred from different sites in all four cases. In cases with IR, late recurrent bleeding was confirmed in 6 of 19 cases (31.6%). Although IR/surgery is an effective hemostatic method for severe bleeding, it does not prevent late recurrent bleeding. Therefore, in cases where endoscopic hemostasis is difficult, less invasive IR should be considered first before surgery. Surgery should be used as only the last resort if hemostasis is not successful. When performing surgery, resection should be minimized, and elective surgery should be chosen because of the high risk of mortality associated with emergency colectomy.^31^

There are several limitations of the study. First, this was a retrospective single‐center study. Second, the number of patients who required IR/surgery was limited. Third, our study did not consider endoscopic hemostasis methods. According to the report by Ishii et al., compared to the clip method, the EBL method has significantly less use of IR.^27^ However, in the present study, the clip method was mainly used for endoscopic hemostasis, and the EBL method was used in only 18 patients. Thus, in the future, we need to conduct a multifacility study with a large number of patients, taking into consideration endoscopic hemostasis methods. Moreover, because severe adverse events have been reported for EBL, such as delayed perforation,^32^, ^33^ it is important to conduct a multifacility examination to consider its safety.

In conclusion, the independent risk factors for IR/surgery were state of shock with a blood pressure of ≤90 mmHg, positive extravasation on CT, two or more episodes of recurrent bleeding, and right colon as the source of bleeding. IR/surgery is an effective hemostatic method for CDB when endoscopic hemostasis is difficult; however, it needs to be noted that late recurrent bleeding cannot be prevented.

## Declaration of conflict of interest

Drs Yoshinori Sato, Hiroshi Yasuda, Yusuke Nakamoto, Hirofumi Kiyokawa, Masaki Yamashita, Tadateru Maehata, Hiroyuki Yamamoto, Hidefumi Mimura, and Fumio Itoh have no conflicts of interest or financial ties to disclose.
